# Diet Diversity Is Associated with Beta but not Alpha Diversity of Pika Gut Microbiota

**DOI:** 10.3389/fmicb.2016.01169

**Published:** 2016-07-27

**Authors:** Huan Li, Tongtong Li, DeAnna E. Beasley, Petr Heděnec, Zhishu Xiao, Shiheng Zhang, Jiabao Li, Qiang Lin, Xiangzhen Li

**Affiliations:** ^1^Key Laboratory of Environmental and Applied Microbiology, Environmental Microbiology Key Laboratory of Sichuan Province, Chengdu Institute of Biology, Chinese Academy of SciencesSichuan, China; ^2^University of Chinese Academy of SciencesBeijing, China; ^3^Department of Biological Sciences, North Carolina State UniversityRaleigh, NC, USA; ^4^State Key Laboratory of Integrated Management of Pest Insects and Rodents in Agriculture, Institute of Zoology, Chinese Academy of SciencesBeijing, China

**Keywords:** diet, gut microbiota, plateau pikas, alpha diversity, beta diversity

## Abstract

Wild mammals often consume different food sources as they become geographical available. This change in diet composition is likely to influence the gut microbial community, yet it remains unclear what the relationship looks like—particularly in small herbivores—under natural conditions. We used DNA sequencing approaches to characterize the diet composition and gut microbial community of wild plateau pikas (*Ochotona curzoniae*) collected from three altitudes. We tested if diet and gut microbiota composition changes across altitudes, and the relationship between diet diversity and gut microbiota diversity. Our results showed that altitude significantly influences the composition of diet and gut microbial communities. Notably, the alpha diversity (Shannon diversity and observed OTUs) of individual diet was not significantly correlated with that of gut microbiota, whereas the beta diversity (Jaccard and Bray-Curtis dissimilarity) of diet was positively correlated with that of gut microbiota. Our study is the first time to highlight the relationship between diet and gut microbiota composition in wild pikas on the Qinghai-Tibet Plateau. It suggests that the species richness within individual gut microbiota does not linearly increase with diet diversity, whereas those individuals that are more similar in diet composition harbor more similar gut microbiota.

## Introduction

Mammalian digestive systems harbor diverse and complex microbial communities that play an important role in shaping host health and function (Tremaroli and Backhed, 2012). To date, severe changes in the gut microbiota have been linked to host metabolic dysfunction and diseases, including obesity, diabetes, cardiovascular disease, and cancer (Gagniere et al., 2016). Understanding how host and environmental factors regulate the composition and diversity of gut microbiota is a key step in assessing mammalian health (Bolnick et al., 2014; Carmody et al., 2015; Pérez-Cobas et al., 2015; Ussar et al., 2015).

Diet is considered to be one of most important environmental factors that influence the assembly of gut microbiota (Turnbaugh et al., 2008; Muegge et al., 2011; Carmody et al., 2015; Pérez-Cobas et al., 2015). Much of what we know about the diet-microbiota relationship comes from studies using artificial diets to assess the effects of single nutrient component, such as high fiber vs. low fiber (Tap et al., 2015), or linear univariate values, such as caloric intake (Zhang et al., 2013). These controlled studies provide insight into how a specific aspect of an organism's diet influences the gut microbiota. However, they do not account for feeding behaviors under natural conditions where organisms are likely to eat a variety of different foods (Baxter et al., 2015) to meet nutritional demands. In fact, in nature, most individual animals consume a variety of food species, rather than focusing on a single diet. Some individuals may have individualized diets or food preferences in a given natural population. In other words, host individuals can differ not only in food species they eat, but also food diversity. It has been reported that gut microbial diversity is a new biomarker of health and metabolic capacity (Clarke et al., 2014). If food diversity is associated with gut microbial diversity, we can improve the host health and metabolic capacity by adjusting food diversity in humans and animals. Only one recent study showed diet mixing had non-additive effects on microbial diversity in fish (Bolnick et al., 2014). Yet, to date, little is known about the relationship between diet diversity and gut microbial diversity in wild mammals, such as herbivores, because it is difficult to accurately evaluate the diet composition.

Diet composition of wild herbivores in natural environments has been studied using two traditional methods, including direct observation (Fan et al., 1995) and microhistology (Liu et al., 2009), in which plant parts from stomach contents were visually identified. These methods have some certain limitations. Direct observation requires high visibility and rare or uncommon plant species are easily overlooked. Microhistology requires intensive efforts and is often imprecise or inaccurate (Carriere, 2002). DNA metabarcoding has been demonstrated to outperform these traditional methods in many respects (Soininen et al., 2009; Pompanon et al., 2012; Newmaster et al., 2013), and can prove at least good at revealing dietary plant species more accurately (Pompanon et al., 2012). Thus, DNA metabarcoding technique can be used to more accurately assess the diet diversity of herbivores and further explore the relationship between diet diversity and gut microbial diversity.

The plateau pika is a small, herbivorous mammal whose range spans throughout the Qinghai-Tibet Plateau, which is a high-altitude steppe ecosystem located more than 3000 m above sea level (ASL) (Luo et al., 2008). The pika is known to feed on various plants available on the plateau (Jiang and Xia, 1985). Different plant communities are distributed along the altitudinal cline on the Qinghai-Tibet Plateau, thus it is likely the pika may feed on different food sources across altitudinal sites.

Here, using plateau pika as a model, we address two key questions:(i) whether altitude influences the composition and structure of diet and gut microbiota? (ii) Whether individual's diet diversity is associated with gut microbial diversity? We found that gut microbial species does not linearly increase with diet species, whereas those individuals that are more similar in diet composition harbor more similar gut microbiota. These results have important implications for understanding diet-microbiota relationship in wild mammals.

## Materials and methods

### Sample collection

All animal experiments and care procedures were consistent with the provision of the Institution of Animal Care and the Ethics Committee of Chengdu Institute of Biology, Chinese Academy of Sciences. Plateau pikas were captured from three different altitudinal sites on the Qinghai-Tibet Plateau, including Guoluo (4331 m ASL) (*n* = 11), Wangjiaxiang (3856 m ASL) (*n* = 8), and Xiaderi (3694 m ASL) (*n* = 5). Pikas were immediately euthanized, and then the stomach and cecal contents were immediately collected and frozen in a −20°C portable freezer. All the samples were transferred to the laboratory within 24 h and stored at −40°C prior to diet and microbiota analysis. The detailed information of 48 samples (24 animal individuals) is listed in Table S1. The plant cover per site was measured in each site according to the Hogan (2010) method. Ten quadrats were randomly selected on each site. Within each quadrat (50 × 50 cm), the plant canopy cover by species was measured with subdecimeter2 resolution using a gridded frame (Davidson and Lightfoot, 2006). In addition, we identified the plant community in each altitudinal site based on morphological characteristics, although we acknowledged that our investigation may not capture all plant species in each site. Species accumulation curves were used to evaluate the plant community diversity in each altitudinal site.

### DNA extraction, PCR amplification, and high-throughput sequencing

Total DNAs of stomach and cecal contents were extracted using Ezup genomic DNA extraction kit for soil (Sangon Biotech, China). For diet analysis, we used ITS3_KYO2F/ITS4R (Toju et al., 2012) primer pairs for the amplification of eukaryotic ITS gene from stomach contents. For the analysis of gut microbiota, we used 515F/909R primer pairs for the amplification of the microbial 16S rRNA gene from cecal contents (Tamaki et al., 2011). The forward primers containing unique 12-bp barcodes were used to tag each PCR product, which allowed us to split sequences to each sample. The 25 μL reactions were carried out in duplicate using 10 ng DNA template, 1x PCR buffer, 1.5 mM MgCl2, each deoxynucleoside triphosphate at 0.4 μM, each primer at 1.0 μM and 0.25 U of Ex Taq (TaKaRa, Dalian). Thermal cycler conditions were: an initial step at 94°C for 3 min, followed by 30 cycles of 94°C for 40 s, 53°C (ITS amplification) or 56°C (16S amplification) for 60 s, and 72°C for 60 s, and a final extension at 72°C for 10 min. After PCR amplification, duplicate PCR runs were combined and PCR products were purified using SanPrep DNA Gel Extraction Kit (Sangon Biotech, China). After equimolar pooling of PCR products, the sequencing samples were prepared using TruSeq DNA kit according to manufacturer's instructions. The purified library was diluted, denatured, re-diluted, mixed with PhiX (equal to 30% of final DNA amount) as described in the Illumina library preparation protocols. Amplicon libraries were sequenced using Illumina Miseq platform (MiSeq Reagent Kit V.2, 500 cycles) at the Environmental Genome Platform of Chengdu Institute of Biology, Chinese Academy of Sciences.

Raw sequences were processed and analyzed using QIIME Pipeline-Version 1.7.0 (http://qiime.org/tutorials/tutorial.html). All reads were trimmed and then assigned to each sample based on their unique barcodes. The two paired-end reads were merged using the FLASH-1.2.8 software (Magoc and Salzberg, 2011). The merged sequences with high quality (reads length >300 bp, without ambiguous base “N”, and average base quality score >30) were used for the following analysis.

### 16 S rRNA and ITS sequences analysis

The filtering and processing of 16S rRNA sequences were described previously (Li et al., 2016). Briefly, after removing chloroplasts and chimeras, All the reads were clustered into operational taxonomic units (OTUs) at a 97% sequence identity using CD-HIT (Li and Godzik, 2006). Because archaeal sequences only accounted for a very small fraction of total reads (<0.01%) in pika guts, we only focused on bacterial microbiota. Thus, those sequences not classifying to bacteria (Eukaryota and Archaea lineages) were removed. Singleton OTUs were also filtered out. Representative sequences for each OTU were picked according to the command line of QIIME script “pick_rep_set.py” (http://qiime.org/scripts/pick_rep_set.html). Thereafter, the sequences were aligned to the Greengenes 13_8 reference database (DeSantis et al., 2006) using PyNAST. The representative sequences of the aligned 16 S rRNA gene sequences were classified through the Ribosomal Database Project classifier (Wang et al., 2007). To compare samples with different sequences, all samples were rarefied to the same number of reads (5528 sequences). To evaluate alpha diversity of bacterial communities, Shannon diversity and observed OTUs were calculated. To assess beta diversity, principal coordinate analysis was performed based on the Jaccard index to represent composition (Jaccard, 1912) and Bray-Curtis index to represent structure (Bray and Curtis, 1957). The Jaccard index was used to compare community similarity based on presence/absence of OTUs, and the Bray-Curtis dissimilarity matrix was used to compare community similarity based on OTU abundance.

For the analysis of ITS sequences, putative chimeras from these reads were identified and excluded using the Uchime algorithm (Edgar et al., 2011), and then were clustered into operational taxonomic units (OTUs) at a 97% identity threshold using CD-HIT (Li and Godzik, 2006). Singleton OTUs were removed. Representative sequences for each OTU were subjected to similarity searches using the BLAST program (Altschul et al., 1997). In addition to plant taxa, our data also included some eukaryotic taxa, such as Alveolata, fungi and Metazoa, whose members would also make up a meaningful portion of the host's diet (Baxter et al., 2015). To minimize the detrimental effects of uneven sampling, these samples were rarefied to the same number of sequences (1670 sequences). The calculation method of alpha diversity and beta diversity were consistent with those of bacterial communities described above.

### Statistical analysis

Because the sample size of each group was uneven, analysis of similarity (ANOSIM) (Dill-McFarland et al., 2016) was used to evaluate whether gut microbiota and diet composition were significantly different across altitudes, animal weight, or sex. ANOSIM analysis was implemented using “anosim” in the R package Vegan (Warton et al., 2012). In addition, redundancy analysis (RDA) of principal coordinates was used to assess the significance of altitude, animal weight or sex using “anova.cca” in the Vegan package. In order to understand the relationship between some specific gut microbes and certain diet or altitude, redundancy analysis was performed and the corresponding biplot was visualized using CANOCO 5 (Microcomputer Power, Ithaca, NY, USA). Differences in relative abundances of taxonomic units among altitudes were tested using one-way-analysis of variance (ANOVA).

Spearman's rank correlation was used to test whether alpha diversity of individual diet was significantly related to corresponding alpha diversity of pika microbial communities (i.e., Shannon diversity of diet and Shannon diversity of gut microbiota, or observed OTUs of diet and observed OTUs of gut microbiota). To test whether beta diversity of diet was correlated with beta diversity of gut microbial communities, we compared Jaccard dissimilarity or Bray-Curtis dissimilarity for diet with the gut microbiota using Mantel tests and Spearman correlations (using Vegan v.2.2-0 in R Core Team 2014). In other words, we tested whether host individuals that were more dissimilar to each other in diet composition also harbored more dissimilar gut microbiota.

## Results

### Vegetation investigation in each altitudinal site

We investigated the plant community in each sampling site. The plant diversity across altitudes did not vary significantly (Figure S1). The dominant plant species across sites was all *Kobresia humilis*, and the plant community in 3694 m ASL was more similar to those in 3856 m ASL than in 4331 m ASL (Figure S2). Only three plant species (*Aconitum szechenyianum, Lagotis gaertn*, and *Stipa capillata*) were different between 3694 and 3856 m ASL, whereas seven plant species (*Astragalus* sp., *Carex moorcraftii, Gueldenstaedtia stenophylla, Kobresia pygmae, Pleurospermum camtschaticum, Scirpus distigmaticus*, and *Thalictrum petaloideum*) showed differences between 3856 m and 4331 m ASL, and 10 plant species (*A. szechenyianum, Astragalus* sp., *C. moorcraftii, G. stenophylla, K. pygmae, L. Gaertn, P. camtschaticum, S. capillata, S. distigmaticus* and *T. petaloideum*) showed differences between 3694 m and 4331 m ASL (Table S2).

### Diet composition and structure across altitudinal sites

**Figure 1:**
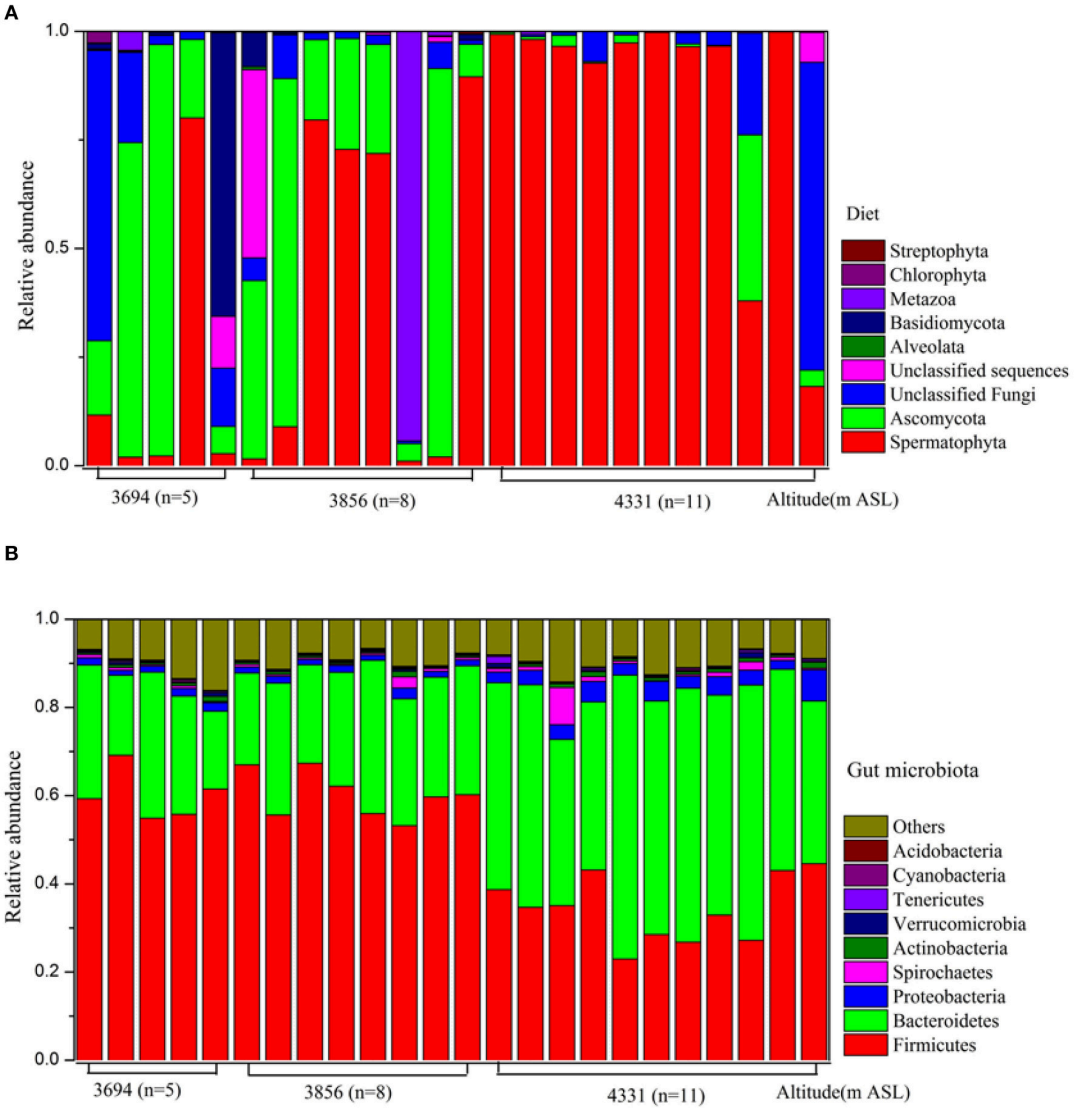
**The composition of diet and gut microbiota at phylum level. (A)** Diet. **(B)** Gut microbiota.

Using ITS sequencing, 56.77% of all sequences were affiliated with plant taxa, including the phyla Spermatophyta (56.63%), Chlorophyta (0.11%), and Streptophyta (0.03%). 36.22% sequences were identified as fungi, which consisted of Ascomycota (22.85%) and Basidiomycota (3.25%) and unclassified fungi (10.12%). Other diet composition included Metazoa (4.27%) and Alveolata (0.04%). OTU-level rarefaction curves of the Goods coverage have reached a plateau (Figure S3), indicating that we have captured most of diet species in pika guts. The diet composition of each animal individual was visualized in Figure 1A.We found that the relative abundances of Spermatophyta were significantly different across altitudes (*P* < 0.05, Table S3).

At genus level, 11 most dominant dietary plant (mean relative abundance >1%) included *Oxytropis, Trachydium, Pedicularis, Ranunculus, Kobresia, Astragalus, Halerpestes, Koenigia, Draba, Triglochin, and Sophora*. The relative abundances of these genera exhibited a wide variation in different altitudinal sites (Table S3). At OTU level, we found that *Oxytropis kansuensis* (12.5%), *Trachydium paradoxum* (6.24%), *Ranunculus brotherusii* (6.24%), *Pedicularis peduncularis* (5.42%), *Kobresia humilis* (3.47%), *Halerpestes cymbalaria* (2.46%), *Astragalus mongholicus* (1.44%), *Triglochin maritime* (1.1%), *Sophora flavescens* (1%) were the main dietary plant species in pikas. Most of these plant species were present in the surrounding habitats in each site (Table S2).

For alpha diversity analysis, we calculated the mean Shannon diversity and observed OTUs of the diet in each sites (Table S4). The Shannon diversity of diet showed no significant differences across altitudinal sites (*P* > 0.05), but the observed OTUs was lower in 4331 m ASL than those in 3856 m and 3694 m ASL (*P* < 0.05). In particular, the Shannon diversity and observed species of dietary plant were not significantly different across sites (*P* > 0.05).

**Figure 2:**
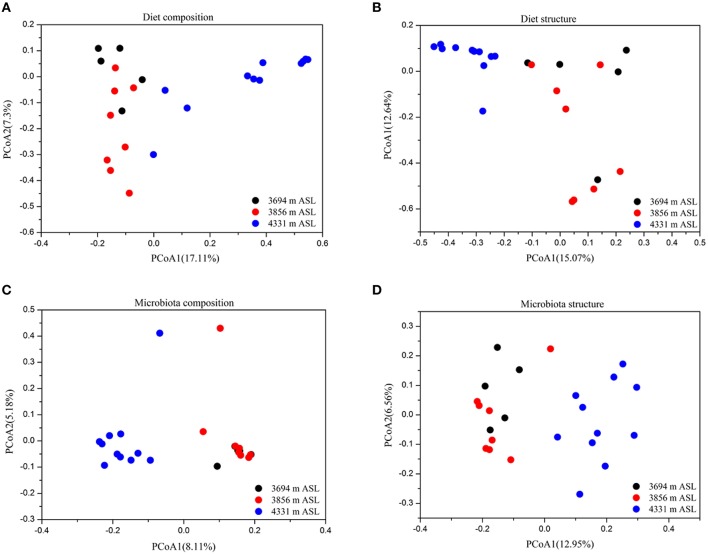
**Principal coordinates analysis showing the composition (Jaccard index) and structure (Bray-Curtis index) of diet and gut microbiota across sites. (A)** Diet composition. **(B)** Diet structure. **(C)** Microbiota composition. **(D)** Microbiota structure.

Principal coordinates analysis showed that the diet composition (Jaccard, ANOSIM *r* = 0.512, *P* < 0.001; Figure 2A) and structure (Bray-Curtis, ANOSIM *r* = 0.58, *P* < 0.001; Figure 2B) were significantly different among sites. However, animal sex and weight had no significant effects on the diet composition and structure (ANOSIM, both *P* > 0.05). Redundancy Analysis (RDA) confirmed that the composition and structure of diet were affected by altitude (*F* = 6.81, *P* < 0.001; *F* = 5.46, *P* < 0.001), while weight and sex had no significant impacts on the diet (*P* > 0.05).

### Gut microbial composition and structure across altitudinal sites

Using 16S rRNA sequencing, we found that the dominant phyla (mean relative abundance>1%) in pikas were Firmicutes (48.30%), Bacteroidetes (36.81%), Proteobacteria (2.50%), and Spirochaetes (1.04%). Other rare phyla (mean relative abundance <1%) included Actinobacteria, Verrucomicrobia, Tenericutes, Cyanobacteria, and Acidobacteria (Figure 1B). The relative abundances of Firmicutes, Bacteroidetes and Proteobacteria in 4331 m ASL were significantly different than those in 3694 and 3856 m ASL (Table S5). The phylum Firmicutes comprised primarily the families Ruminococcaceae and Lachnospiraceae, while Bacteroidetes comprised the families S24-7, Prevotellaceae and Paraprevotellaceae. Despite a few differences in different sites, *Prevotella, Oscillospira, Ruminococcus*, and *YRC22* were the predominant bacterial genera (Table S5).

Similarly, we calculated the Shannon diversity and observed OTUs of gut microbiota in each site (Table S4). OTU-level rarefaction curves of the Goods coverage across all samples has reached stable values (Figure S3), indicating that most of the gut microbial diversity had already been captured in our results despite the possibility to detect rare new phylotypes with additional sequencing depth. The Shannon diversity indices were similar among different sites (*P* > 0.05), whereas the gut microbiota in 4331 m ASL had a lower observed species than those in 3694 and 3856 m ASL (Table S6).

For beta diversity analysis, we found that altitude significantly influenced the gut microbiota composition (Jaccard, ANOSIM *r* = 0.699, *P* < 0.001; Figure 2C) and structure (Bray-Curtis, ANOSIM *r* = 0.56, *P* < 0.001; Figure 2D). However, the composition and structure of pika gut microbiota were not influenced by animal sex and weight (ANOSIM, *P* > 0.05). The results of RDA also showed that the composition and structure of gut microbial communities were affected by altitude (*F* = 2.00, *P* < 0.001; *F* = 3.63, *P* < 0.001, respectively), but sex and weight had no any significant effects on the gut microbiota (*P* > 0.05).

### The correlation between some specific microbes and diet or altitude

**Figure 3:**
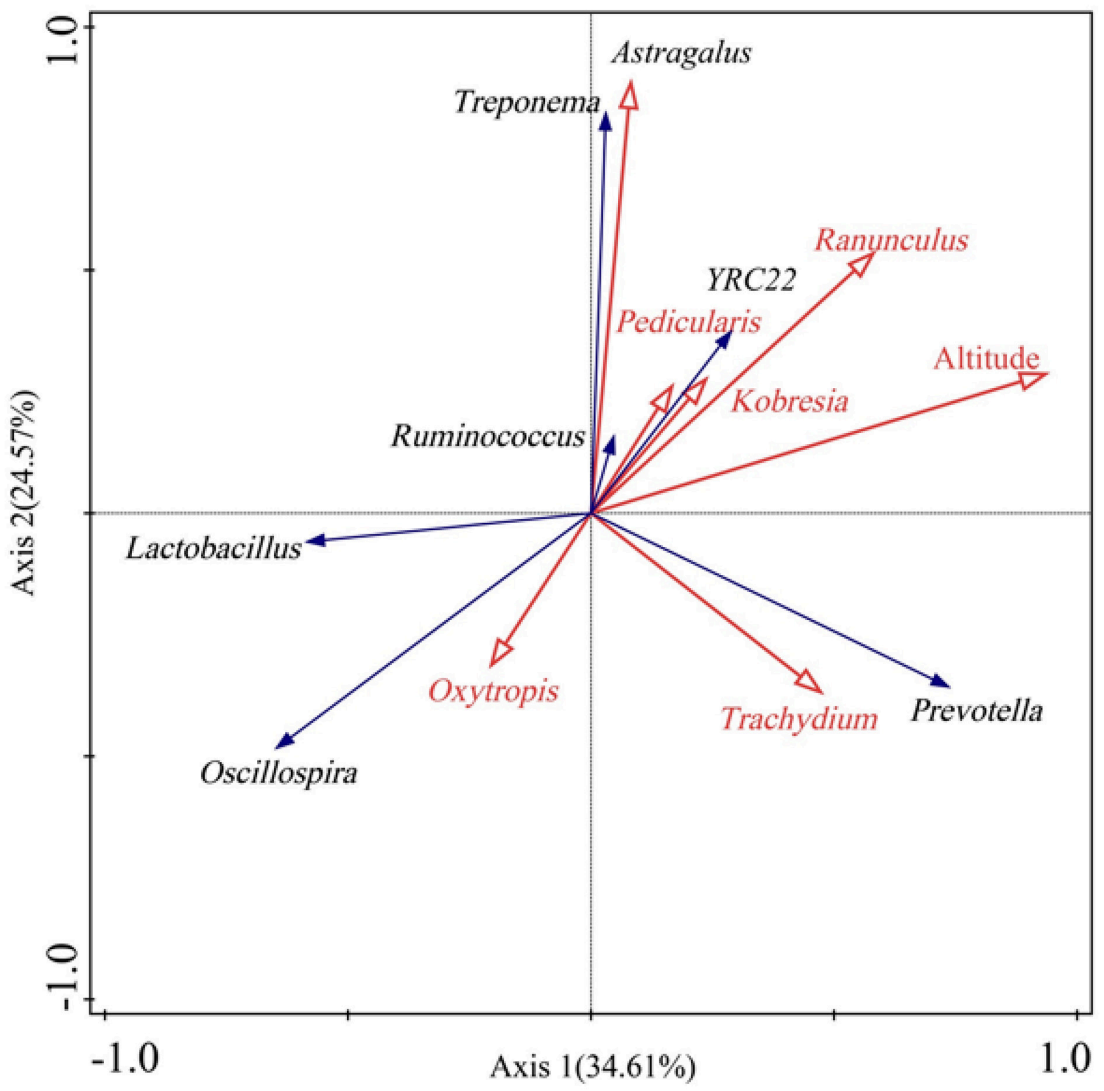
**Biplot of redundancy analysis (RDA) of gut bacterial genera (>0.5%) responding to altitude and certain diet**. Only those dietary plant genera with relative abundances >1.5% were shown.

Redundancy analysis (RDA) showed that some bacterial genera were associated with certain diet (Figure 3). For example, the bacterial genus *Oscillospira* was positively correlated with the plant genus *Oxytropis* in diet. *Prevotella* showed a positive correlation with the plant *Trachydium*. In addition, some gut microbes were related to altitude. For instance, *Prevotella, Ruminococcus*, and *Treponema* showed positive correlations with altitude, while Oscillospira was negatively associated with altitude.

### The relationship between alpha diversity of diet and gut microbiota

**Figure 4:**
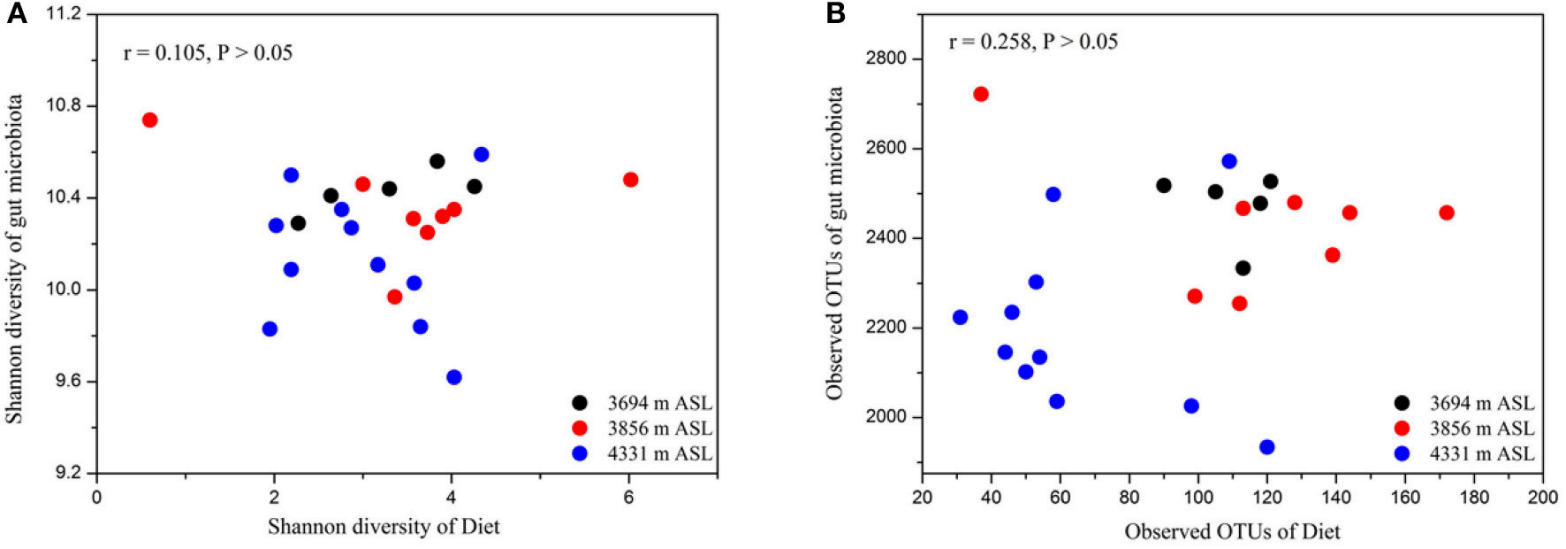
**Relationships between alpha diversity of diet and gut microbiota. (A)** The Shannon diversity. **(B)** The observed OTUs. No relationships were significant (*P* > 0.05 in both cases).

Using spearman correlation analysis, we tested whether measures of microbial alpha diversity were significantly related to corresponding diet alpha diversity measures (i.e., microbial Shannon diversity with diet Shannon diversity, or microbial observed OTUs with diet observed OTUs). We found no significant relationships between microbial alpha diversity measures per individual and any of their corresponding diet diversity measures (Figures 4A,B; both *P* > 0.05). In particular, we sought to determine whether the alpha diversity of dietary plant was associated with that of pika gut microbiota. There were also no significant relationships between the alpha diversity of dietary plant and gut microbiota (Figure S4; both *P* > 0.05).

### The relationship between beta diversity of diet and gut microbiota

**Figure 5:**
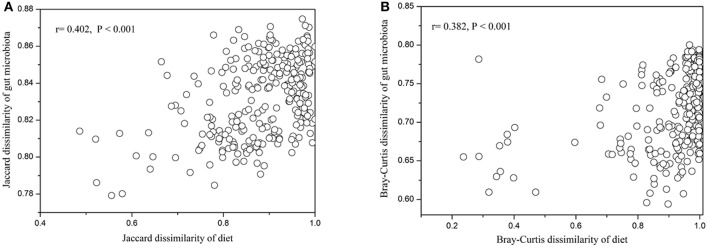
**Relationships between beta diversity for diet and gut microbiota. (A)** Jaccard dissimilarity. **(B)** Bray-Curtis dissimilarity.

In contrast to the lack of correlations for alpha diversity, our results showed significant positive relationships between the beta diversity of diet and gut microbiota across all animal individuals: i.e., individuals that were more distinct in diet composition also harbored more distinct gut microbial communities. This was consistent for composition (Jaccard, *r* = 0.402, *P* < 0.001, Figure 5A), and structure (Bray-Curtis, *r* = 0.382, *P* < 0.001, Figure 5B) of diet and gut microbiota. In particular, we found that the dissimilarity of dietary plant was also positively associated with that of gut microbiota (Jaccard, *r* = 0.369, *P* < 0.001; Bray-Curtis, *r* = 0.333, *P* < 0.001, Figure S5).

## Discussion

In feces or gut contents, some diet components have been degraded and absorbed, and only small DNA fragments remain (Deagle et al., 2006). Thus, we chose stomach contents for diet analysis, because the diet DNA in stomach is more intact than that in feces or gut contents. Our primers have a high coverage for eukaryotes, such as plant and fungi, and these taxa were considered as significant components of animals' diet (Baxter et al., 2015). Although pikas are herbivorous animals, our results showed other non-plant foods (e.g., fungi, Metazoa, and Alveolata) were also possible to be their diet. Using ITS gene sequencing, most of dietary plant can be identified at species level, and the method is more accurate and convenient in the identification of diet composition in wild animals than traditional methods (direct observation and microhistology). However, DNA metabarcoding technique has a limited ability to reveal relative amounts of diets due to variations in DNA content and copy numbers across different eukaryotic taxa (Codron et al., 2007). In addition, the method also has bias introduced by laboratory procedures. For example, primer mismatches may inhibit amplification of some plant species (Pompanon et al., 2012).

Previous studies (traditional methods) showed that the plateau pikas mainly feed on plants belonging to the Cyperaceae and Poaceae families, such as *Elymus nutans, Kobresia* sp., *Carex* sp., and *Poa* sp.(Jiang and Xia, 1985; Liu et al., 1991; Wang et al., 1992). Using DNA metabarcoding technique, our results broaden the scope of diet profiles for plateau pikas on the Qinghai-Tibet Plateau. We found that the family Leguminosae (e.g., *O. kansuensis*) and Ranunculaceae (e.g., *R. brotherusii*) were also dominant plant species available by plateau pikas. Most of these dominant plant species were presented in surrounding habitats, indicating that our results were reliable. Some plant genera were associated with specific bacterial genera, the possible explanation is that the specific nutritional components of the diet lead to the enrichment of these bacteria. However, our results showed that the diet composition had great inter-individual variations, and was influenced by altitudinal sites. The results suggested that, both food resources of pikas' habitat and food preferences of animal individuals may affect the individual diet composition in the wild.

The phyla Firmicutes and Bacteroidetes were the dominant taxa in pika gut, similar to other mammalian systems (Ley et al., 2008). However, we found that an increase in proportion of Bacteroidetes and a decrease in the proportion of Firmicutes along the altitudinal cline (Table S5). At genus level, *Prevotella* and *Ruminococcus* were the dominant identified genera in the pika gut. Some strains of these genera are associated with plant-rich diets, and they have been identified as active microbes, which can express various genes encoding carbohydrate-degrading enzymes (Dai et al., 2014). These functional bacteria were positively associated with altitude, indicating that high-altitude pikas had a higher abundance of cellulose-degrading bacteria, which may help pikas digest plant cellulose more efficiently. The genus *Oscillospira* was the second dominant genus in the gut, and it showed wide variations among different altitudinal sites. This genus has been detected in other herbivores mammals, such as cattle and sheep (Mackie et al., 2003). Some members of *Oscillospira* are associated with fresh forage (Mackie et al., 2003), whereas their functional roles in herbivores need to be further determined.

Our results showed that the gut microbiota composition and structure were influenced by altitude, but not by animal weight or sex. Because food resources were distinct in different altitudinal sites, diet may play major roles in shaping the gut microbial communities. In addition, each pika had an individualized diet profile, which may lead to different gut microbiota composition compared with other individuals even though these animals were located in the same regions.

Thus, we sought to explore whether diet diversity is associated with gut microbial diversity. In general, diet-associated microbes have a wider source accompanied by more diverse foods, so those animals, which consume more diverse foods, may be exposed to and carry more diverse microbes (Laparra and Sanz, 2010). On the other hand, microbiota composition also relies on nutrients in the gut, thus diverse diets may increase the alpha diversity of gut microbiota by providing more diverse nutrients. These hypotheses were not supposed by our data, which showed the alpha diversity (Shannon diversity and observed OTUs) of diet was not associated with that of pika gut microbiota. Our results were consistent with those of Bolnick et al. (2014), who demonstrated that diverse diets had non-additive effects on microbial diversity in fish gut (Bolnick et al., 2014). We enumerate some hypotheses for further testing in future. First, some microbes have a broad spectrum of nutrients in the gut environments, and they have gained competitive advantages and become those dominant microbes in the gut. In contrast, some rare microbes have a specific spectrum of nutrients and are unable to persist in the host gut. Consequently, the alpha diversity of gut microbiota does not linearly increase with more diverse foods. Second, each food might contain chemical inhibitors that influence the presence or growth of certain microbes. Thus diet diversity may not increase linearly with gut microbial diversity. Third, food has indirect effects on host physiology and immunity, which may regulate the diversity of gut microbiota. For example, food affects the production of bile acids, which is thought to protect the gut from exotic microbe species in humans (Ridlon et al., 2015).

In contrast to the lack of alpha diversity, we found that positive correlations between the beta diversity of gut microbiota and diet. The results suggested that individuals that are more similar in diet harbor more similar gut microbiota in natural environments. Previous studies focused on the effects of a broad level of feeding habitats (e.g., herbivores, omnivores, and carnivores) that influence gut microbiota (Ley et al., 2008), we identified the diet profiles more accurately at species level. The results we present have important implications for treating dysbiosis, as we can regulate the atypical gut microbiota by adjusting food species and structure.

To our knowledge, this is the first report to investigate the relationship between diet and gut microbiota in wild mammals using next-generation sequencing. We offer a novel method to explore relationship between the diet and gut microbiota in wild herbivores. However, it remains to be determined whether diet diversity is associated with gut microbial diversity in other host species. Our study focused on the correlation rather than causal relationship between diet and gut microbiota. In order to prove a causal link between diet and gut microbial community, strict control experiments in laboratory are necessary. In addition, future research needs to further explore the relationship between diet and gut microbiota function in humans and animals.

## Sequence data accession number

The original ITS sequence data are available at the European Nucleotide Archive by accession number PRJEB13006 (http://www.ebi.ac.uk/ena/data/view/PRJEB13006). The original 16S rRNA sequence data are available at the European Nucleotide Archive by accession number PRJEB13008 (http://www.ebi.ac.uk/ena/data/view/PRJEB13008).

## Author contributions

HL designed research; HL, TL, PH, SZ, JL, and QL contributed to experimental work; HL performed the data analysis and wrote the manuscript. DB, ZX, and XL revised the manuscript.

### Conflict of interest statement

The authors declare that the research was conducted in the absence of any commercial or financial relationships that could be construed as a potential conflict of interest.
