# Supplementary material for: Diet Diversity Is Associated with Beta but not Alpha Diversity of Pika Gut Microbiota
Source: Front Microbiol. 2016 Jul 27;7:1169. doi: 10.3389/fmicb.2016.01169 (PMC4961685; doi:10.3389/fmicb.2016.01169)
Supplement: Supplementary file 7 [file Image1.PDF]

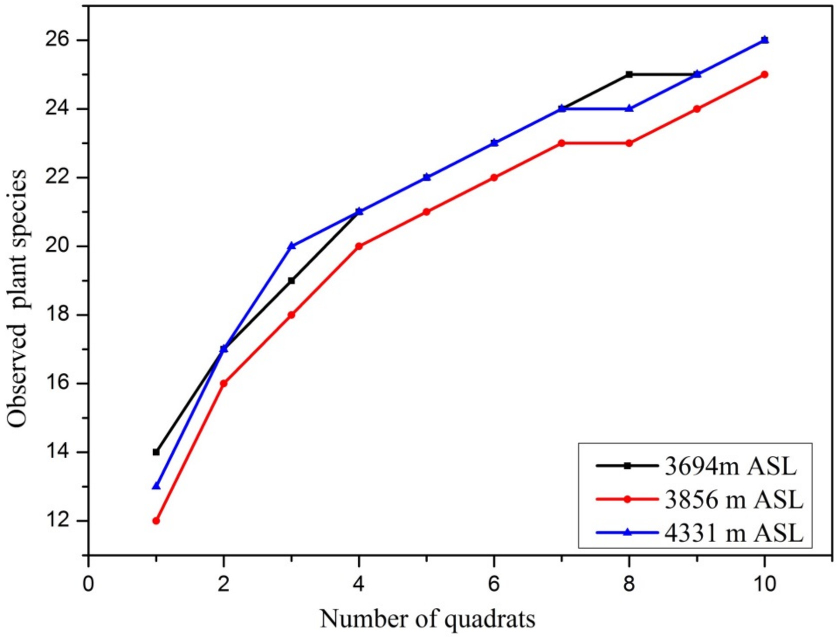

Figure S1 Accumulation curves of plant species in the habitats of pikas across altitudinal sites
