# Supplementary material for: Diet Diversity Is Associated with Beta but not Alpha Diversity of Pika Gut Microbiota
Source: Front Microbiol. 2016 Jul 27;7:1169. doi: 10.3389/fmicb.2016.01169 (PMC4961685; doi:10.3389/fmicb.2016.01169)
Supplement: Supplementary file 8 [file Image2.PDF]

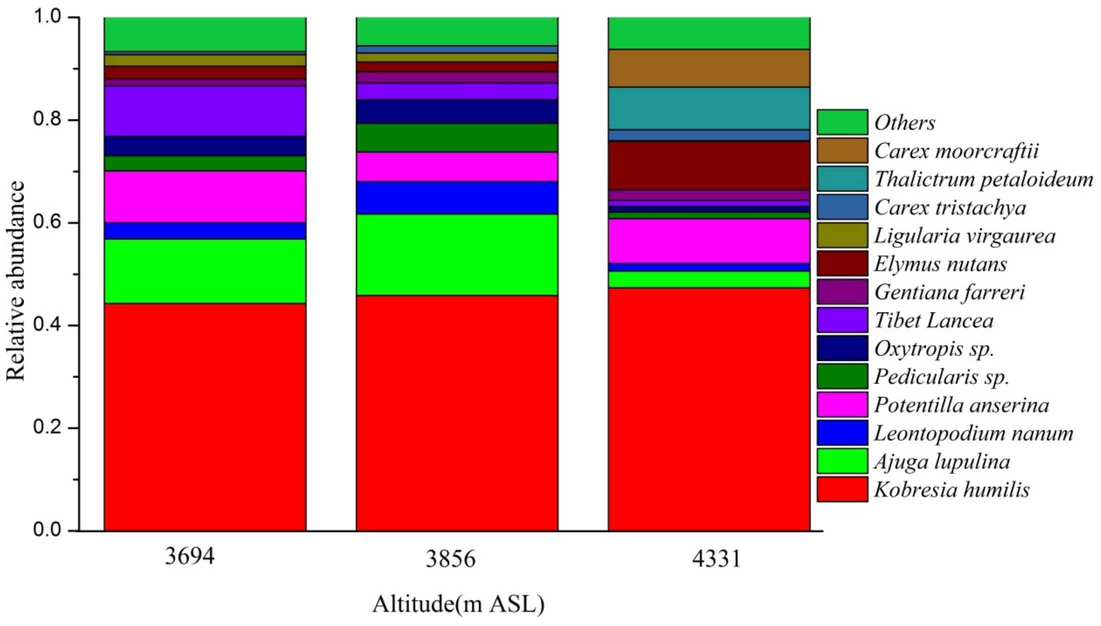

Figure S2 The composition of the plant cover in different altitudinal sites. Only those plant species with relative abundance > 1% are shown.
