# Supplementary material for: Diet Diversity Is Associated with Beta but not Alpha Diversity of Pika Gut Microbiota
Source: Front Microbiol. 2016 Jul 27;7:1169. doi: 10.3389/fmicb.2016.01169 (PMC4961685; doi:10.3389/fmicb.2016.01169)
Supplement: Supplementary file 9 [file Image3.PDF]

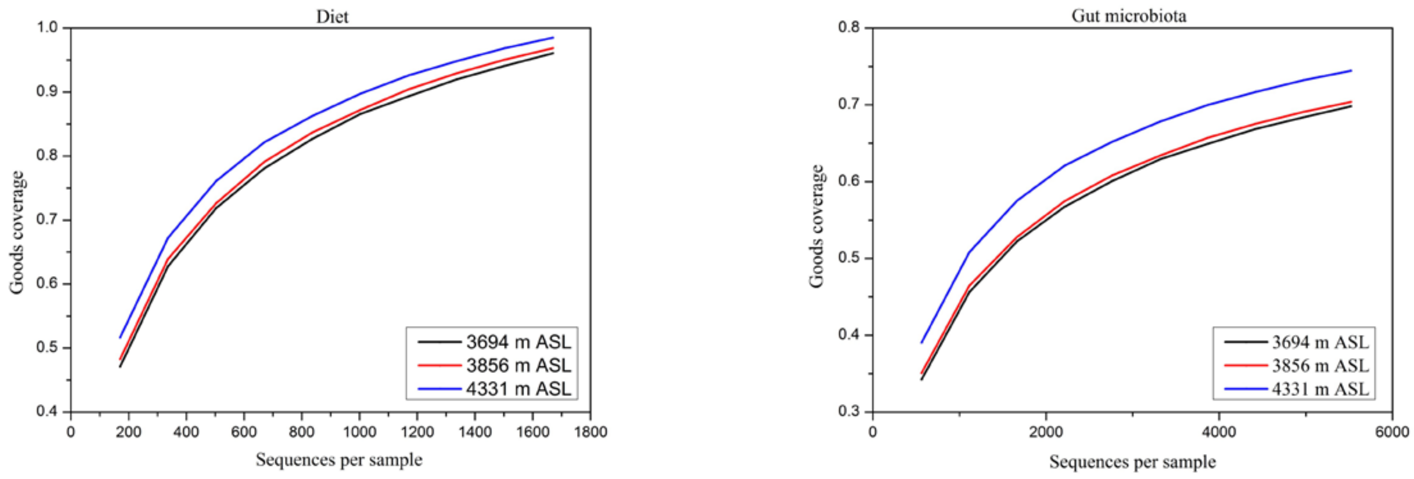

Figure S3 OTU-level rarefaction curves of Goods coverage in diet and gut microbiota of pikas across altitudinal sites
