# Supplementary material for: Diet Diversity Is Associated with Beta but not Alpha Diversity of Pika Gut Microbiota
Source: Front Microbiol. 2016 Jul 27;7:1169. doi: 10.3389/fmicb.2016.01169 (PMC4961685; doi:10.3389/fmicb.2016.01169)
Supplement: Supplementary file 10 [file Image4.PDF]

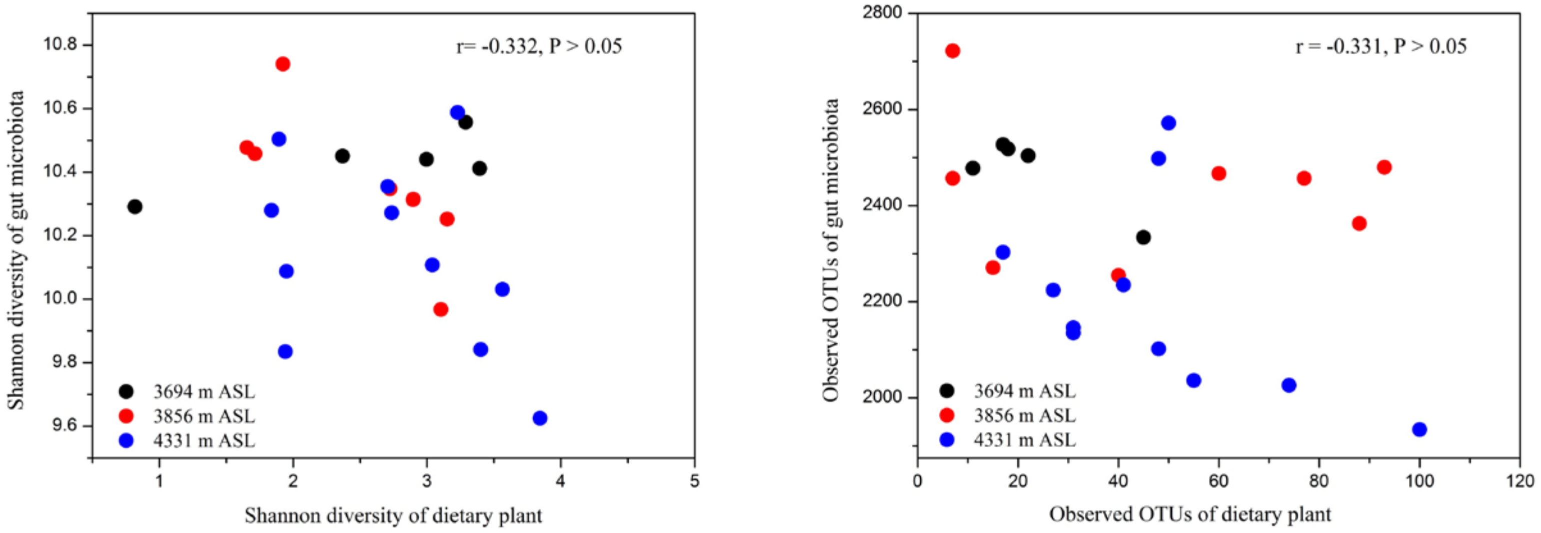

Figure S4 Relationships between alpha diversity of dietary plant and gut microbiota. (a) The Shannon diversity. (b) The observed OTUs. No relationships were significant (P>0.05 in both cases).
