# Supplementary material for: Diet Diversity Is Associated with Beta but not Alpha Diversity of Pika Gut Microbiota
Source: Front Microbiol. 2016 Jul 27;7:1169. doi: 10.3389/fmicb.2016.01169 (PMC4961685; doi:10.3389/fmicb.2016.01169)
Supplement: Supplementary file 11 [file Image5.PDF]

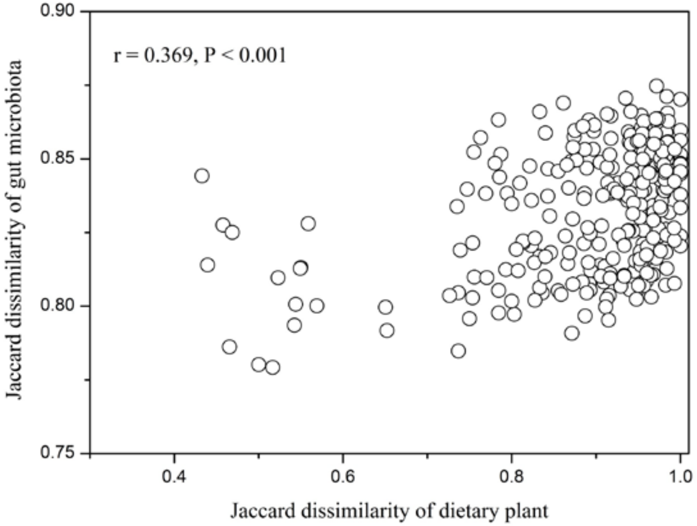

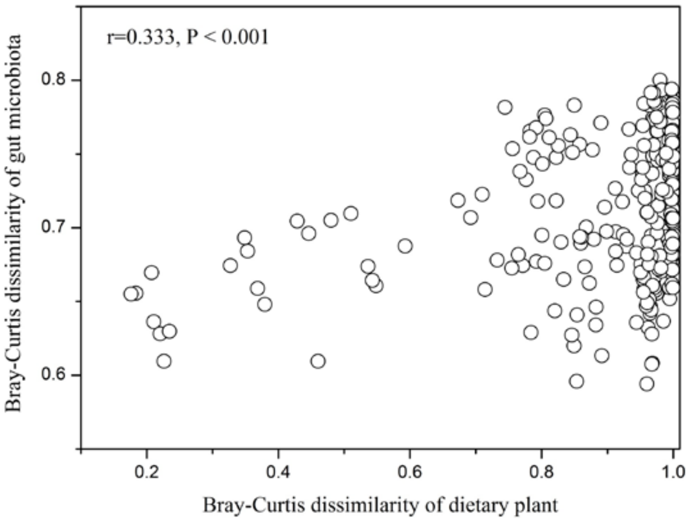

Figure S5 Relationships between beta diversity for dietary plant and gut microbiota. (a) Jaccard dissimilarity. (b) Bray-Curtis dissimilarity.
